# Infrared imaging of magnetic octupole domains in non-collinear antiferromagnets

**DOI:** 10.1093/nsr/nwad308

**Published:** 2023-12-04

**Authors:** Peng Wang, Wei Xia, Jinhui Shen, Yulong Chen, Wenzhi Peng, Jiachen Zhang, Haolin Pan, Xuhao Yu, Zheng Liu, Yang Gao, Qian Niu, Zhian Xu, Hongtao Yang, Yanfeng Guo, Dazhi Hou

**Affiliations:** International Center for Quantum Design of Functional Materials (ICQD), School of Emerging Technology, University of Science and Technology of China, Hefei 230026, China; College of Mathematics and Physics, Qingdao University of Science and Technology, Qingdao 266061, China; School of Physical Science and Technology, ShanghaiTech University, Shanghai 201210, China; ShanghaiTech Laboratory for Topological Physics, ShanghaiTech University, Shanghai 201210, China; International Center for Quantum Design of Functional Materials (ICQD), School of Emerging Technology, University of Science and Technology of China, Hefei 230026, China; Department of Physics, University of Science and Technology of China, Hefei 230026, China; International Center for Quantum Design of Functional Materials (ICQD), School of Emerging Technology, University of Science and Technology of China, Hefei 230026, China; Department of Physics, University of Science and Technology of China, Hefei 230026, China; International Center for Quantum Design of Functional Materials (ICQD), School of Emerging Technology, University of Science and Technology of China, Hefei 230026, China; Department of Physics, University of Science and Technology of China, Hefei 230026, China; International Center for Quantum Design of Functional Materials (ICQD), School of Emerging Technology, University of Science and Technology of China, Hefei 230026, China; Department of Physics, University of Science and Technology of China, Hefei 230026, China; International Center for Quantum Design of Functional Materials (ICQD), School of Emerging Technology, University of Science and Technology of China, Hefei 230026, China; Department of Physics, University of Science and Technology of China, Hefei 230026, China; International Center for Quantum Design of Functional Materials (ICQD), School of Emerging Technology, University of Science and Technology of China, Hefei 230026, China; Department of Physics, University of Science and Technology of China, Hefei 230026, China; Department of Physics, University of Science and Technology of China, Hefei 230026, China; CAS Key Laboratory of Strongly-Coupled Quantum Matter Physics, University of Science and Technology of China, Hefei 230026, China; Department of Physics, University of Science and Technology of China, Hefei 230026, China; CAS Key Laboratory of Strongly-Coupled Quantum Matter Physics, University of Science and Technology of China, Hefei 230026, China; Department of Physics, University of Science and Technology of China, Hefei 230026, China; CAS Key Laboratory of Strongly-Coupled Quantum Matter Physics, University of Science and Technology of China, Hefei 230026, China; School of Physical Science and Technology, ShanghaiTech University, Shanghai 201210, China; Xi’an Institute of Optics and Precision Mechanics of Chinese Academy of Sciences, Xi’an 710119, China; School of Physical Science and Technology, ShanghaiTech University, Shanghai 201210, China; ShanghaiTech Laboratory for Topological Physics, ShanghaiTech University, Shanghai 201210, China; International Center for Quantum Design of Functional Materials (ICQD), School of Emerging Technology, University of Science and Technology of China, Hefei 230026, China; Department of Physics, University of Science and Technology of China, Hefei 230026, China

**Keywords:** non-collinear antiferromagnet, domain structure, infrared imaging, anomalous Ettingshausen effect

## Abstract

Magnetic structure plays a pivotal role in the functionality of antiferromagnets (AFMs), which not only can be employed to encode digital data but also yields novel phenomena. Despite its growing significance, visualizing the antiferromagnetic domain structure remains a challenge, particularly for non-collinear AFMs. Currently, the observation of magnetic domains in non-collinear antiferromagnetic materials is feasible only in Mn_3_Sn, underscoring the limitations of existing techniques that necessitate distinct methods for in-plane and out-of-plane magnetic domain imaging. In this study, we present a versatile method for imaging the antiferromagnetic domain structure in a series of non-collinear antiferromagnetic materials by utilizing the anomalous Ettingshausen effect (AEE), which resolves both the magnetic octupole moments parallel and perpendicular to the sample surface. Temperature modulation due to AEE originating from different magnetic domains is measured by lock-in thermography, revealing distinct behaviors of octupole domains in different antiferromagnets. This work delivers an efficient technique for the visualization of magnetic domains in non-collinear AFMs, which enables comprehensive study of the magnetization process at the microscopic level and paves the way for potential advancements in applications.

## Teaser

Infrared imaging employing the anomalous Ettingshausen effect unveils magnetic domain structures in non-collinear antiferromagnets, unlocking new possibilities in spintronics and memory device development.

## INTRODUCTION

The non-collinear antiferromagnet Mn_3_X family has emerged as a significant class of materials in the field of spintronics [1–7]. These materials possess unique physical properties, including the magnetic spin Hall effect [8], the large anomalous Hall/Nernst effects (AHE/ANE) [9–11], the Weyl semimetal points [12] and the chiral domain wall memory effect [13]. Recent progress further demonstrated their electrical manipulation and readout capabilities, making them promising building elements for future memory devices [4,5].

Therefore, the domain structure visualization in a non-collinear antiferromagnet (AFM) is greatly pursued [14–22], which is indispensable for confirming and understanding the magnetic octupole dynamics driven by magnetic field and spin-orbit torque [23–28]. Although the magneto-optical Kerr effect (MOKE) has been successfully used to image domain structures in Mn_3_Sn [14,16], it requires a pristine mirror surface and can image magnetic octupole domains with out-of-plane polarization with only few-nanometer depth [29]. The anomalous Nernst effect, on the other hand, can image the octupole domain with in-plane polarization, but is limited to in-plane observations and is time consuming for large devices due to the scanning approach [15]. Despite early interest, the direct imaging and reconstruction of three-dimensional (3D) rotation of octupole moments, which calls for simultaneous observation of both in-plane and out-of-plane octupole domains, remains elusive.

## RESULTS AND DISCUSSION

Here, we demonstrate an alternative method for imaging the domain structure in non-collinear AFMs. We show that the domain structure can be imaged by the anomalous Ettingshausen effect (AEE) at room temperature, capturing octupole moments both parallel and perpendicular to the sample surface. The visualization of magnetic domains through the AEE was first demonstrated in ferromagnetic metals [30,31]. The method is based on visualizing the spatial distribution of AEE-induced temperature modulations originating from different magnetic octupole domains, and is achieved through lock-in thermography (LIT) [32–34]. Employing the new technique, we achieve observation of the octupole domain structure in Mn_3_Sn and Mn_3_Ge during both in-plane and out-of-plane magnetic reversals and reveal the out-of-plane rotation process of octupole moments in the memory effect of Mn_3_Sn [13].

**Figure 1.**
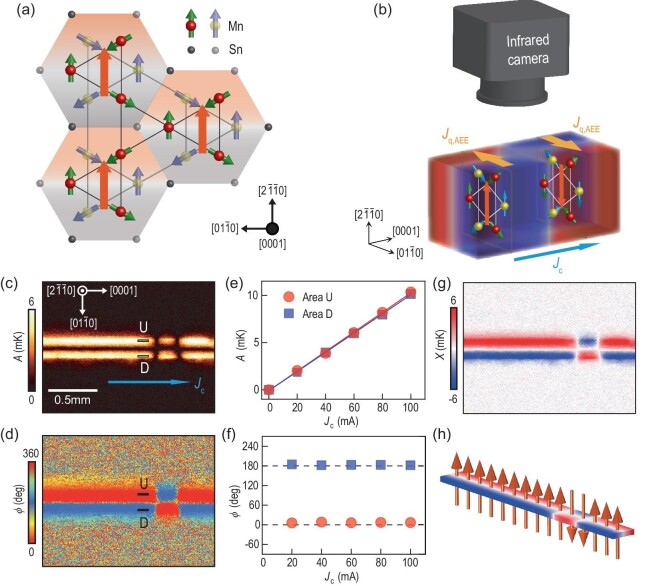
Magnetic structure of Mn_3_Sn and infrared imaging of magnetic domains by AEE. (a) Red and yellow spheres denote Mn atoms on two stacked kagome planes, and the arrows on the atoms represent the moments. The spin texture can be viewed as a ferroic order of a cluster magnetic octupole. The orange arrows represent the direction of the octupole moment. (b) A schematic of infrared imaging of magnetic octupole domains with opposite polarization using AEE. We denote by ${\boldsymbol {\mathrm{J}}}_{\rm c}$ and ${\boldsymbol {\mathrm{J}}}_{\rm q,AEE}$ the applied charge current and heat current generated by AEE, respectively. (c), (d) Lock-in amplitude *A* and phase ϕ images for the $(2\bar{1}\bar{1}0)$ plane of a Mn_3_Sn slab with an out-of-plane easy axis at *J*_c_ = 50 mA along the [0001] direction. (e), (f) The *J*_c_ dependence of *A* and ϕ in the regions of interest on the Mn_3_Sn slab that are defined by two rectangles marked with U and D. The plotted data were obtained by averaging the values in each area, which consists of 28 pixels. (g) Image of $X = A\rm {cos}\phi$. (h) Magnetic octupole domain patterns of the Mn_3_Sn slab revealed by the temperature modulation in (g).

Figure 1(a) shows the magnetic structure of Mn_3_Sn, which hosts a hexagonal crystal structure with *P*6_3_/*mmc* space group. Below its Néel temperature *T*_N_ ≈ 430 K, it exhibits an inverse triangular spin structure of three neighboring Mn moments on its (0001)-plane kagome lattice. This spin structure can be viewed as a ferroic order of a cluster magnetic octupole [35], which is composed of six Mn atoms situated within two stacked kagome planes. The cluster magnetic octupole breaks the time-reversal symmetry macroscopically, which permits a non-zero net Berry curvature in momentum space [36,37], thereby eliciting significant transverse responses, such as the large AHE and ANE [9–11,38,39]. Additionally, the competition between the Dzyaloshinskii–Moriya interaction and single-ion anisotropy results in a small net ferromagnetic moment through spin canting [40], which is essential for manipulating the direction of octupole moments using a magnetic field.

### Magnetic domain imaging by AEE

Figure 1(b) shows the set-up for infrared imaging of magnetic octupole domain structures using AEE. In view of the large ANE in Mn_3_Sn [10,11], a large AEE in Mn_3_Sn is expected by the Bridgman relation [41] between AEE and ANE. AEE can convert a longitudinal electric current ${\boldsymbol {\mathrm{J}}}_{\rm c}$ into a transverse heat current ${\boldsymbol {\mathrm{J}}}_{\rm q,AEE}$ that is dependent on the direction of the octupole moment in Mn_3_Sn. The heat current generates a thermal gradient ∇*T*_AEE_, so the phenomena of AEE can be described as


(1) \begin{eqnarray*}
\nabla T_{{\rm AEE}} = \varepsilon _{{\rm AEE}} ({\boldsymbol {\mathrm{j}}}_{\rm c}\times {\boldsymbol {\mathrm{p}}}),
\end{eqnarray*}

where ${\boldsymbol {\mathrm{j}}}_{\rm c}$ is the charge current density, ${\boldsymbol {\mathrm{p}}}$ is the unit vector of the octupole moment and ε_AEE_ is the anomalous Ettingshausen coefficient. Therefore, by measuring the AEE-induced temperature distribution on the sample surface, one can reveal the underlying octupole domain structures.

In particular, when an in-plane current is applied, the domains with opposite out-of-plane ${\boldsymbol {\mathrm{p}}}$ generate opposite in-plane thermal gradients on the sample surface (top view of Fig. 1(b)), while the domains with opposite in-plane ${\boldsymbol {\mathrm{p}}}$ produce either a cold or hot region on the sample surface (front view of Fig. 1(b)). To capture both scenarios with a much-improved temperature resolution (0.1 mK), LIT is utilized [32–34]. In this work, thermal images of the sample surface are captured while applying a rectangular-wave-modulated charge current with amplitude *J*_c_ and frequency *f* = 16.25 Hz. The use of such current allows for the extraction of thermoelectric effects (∝ *J*_c_) free from the Joule-heating background ($\propto J_{\rm c}^2$) [42,43]. Fourier analyses are performed to extract the first harmonic response of the detected thermal images, yielding lock-in amplitude *A* and phase ϕ images, with the *A* images providing the magnitude of the AEE-induced temperature modulation and the ϕ images providing information on the sign of the temperature modulation and the time delay due to thermal diffusion.

Panels (c) and (d) of Fig. 1 respectively display the *A* and ϕ images for a Mn_3_Sn slab with $(2\bar{1}\bar{1}0)$ oriented surface at zero magnetic field after oscillation demagnetization and *J*_c_ = 50 mA along the [0001] direction. All Mn_3_Sn crystal samples used in this study were grown by bismuth flux method without polishing, from which the Kerr signal cannot be detected probably due to surface oxidization and roughness (see Fig. S1 within the online supplementary material). We observe three clearly distinct temperature-modulation regions: the left and right regions show temperature increase and decrease in the up and down halves, while the middle region shows an opposite pattern, indicating opposite in-plane temperature gradients along $[01\bar{1}0]$. For quantitative analysis, two regions of interest along $[01\bar{1}0]$, denoted U and D, are defined on the Mn_3_Sn slab. In panels (e) and (f) of Fig. 1, we show the *J*_c_ dependence of *A* and ϕ for U and D. The plotted data are obtained by averaging the values in each area. The *A* values are proportional to *J*_c_, while the ϕ values remain unchanged with respect to *J*_c_ and exhibit a 180^○^ shift for the U and D regions, which is in good agreement with the features of AEE with the local ${\boldsymbol {\mathrm{p}}}$ along the $[2\bar{1}\bar{1}0]$ direction.

Figure 1(g) shows the *X* = *A*cos ϕ image calculated from panels (c) and (d). The use of *X* = *A*cos ϕ to represent temperature modulation with sign information is more intuitive and makes it easier to calculate the orientation of octupole domains in the absence of a significant phase delay. Therefore, throughout the following text, we adopt the *X* image to present the spatial distribution of temperature modulation. Taking into account the magnetic anisotropy that tends to align ${\boldsymbol {\mathrm{p}}}$ along the $[2\bar{1}\bar{1}0]$ direction [44–46], these observations strongly suggest that the sample has a domain distribution with upward oriented ${\boldsymbol {\mathrm{p}}}$ on the left and right sides and downward oriented ${\boldsymbol {\mathrm{p}}}$ in the middle. Assuming AEE to be the origin of the thermal patterns, in Fig. 1(h) we plot the magnetic octupole domain patterns of the Mn_3_Sn slab that are revealed by the *X* image in Fig. 1(g).

### Imaging the out-of-plane magnetic reversal of Mn_3_Sn

**Figure 2.**
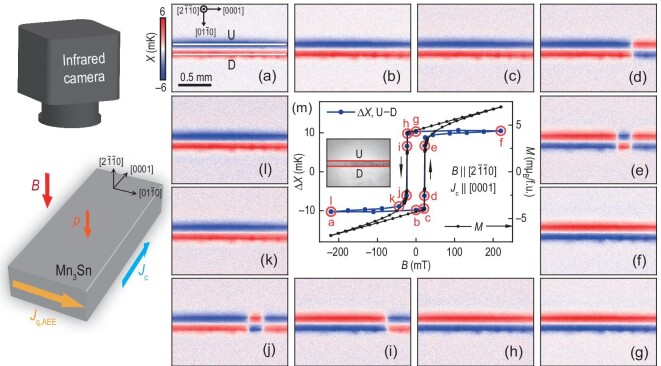
Infrared imaging of magnetic domain patterns in Mn_3_Sn during out-of-plane magnetic reversal. (a)–(l) The *X* images obtained in the out-of-plane field-scan cycle from −220 to 220 mT along $[2\bar{1}\bar{1}0]$ at *J*_c_ = 50 mA along [0001]. (m) Field dependence of Δ*X* calculated from the U and D areas of the Mn_3_Sn slab, where the plotted data were obtained by subtracting the average values on D from U, which consists of 640 pixels. For comparison, the field dependence of magnetization *M* is shown. Inset shows the DC infrared image of the $(2\bar{1}\bar{1}0)$ surface plane of the Mn_3_Sn slab. The thickness and width of the slab are approximately 0.09 and 0.17 mm, with a charge current density of *j*_c_ = 3.3 × 10^3^ A m^−2^.

To verify the validity of AEE for imaging octupole domain structures, we applied it to visualize the domain reversal by external magnetic fields. Panels (a)–(l) of Fig. 2 display a series of *X* images of the same sample as in Fig. 1(c) under the out-of-plane field-scan cycle from −220 to 220 mT along $[2\bar{1}\bar{1}0]$. The applied current *J*_c_ is 50 mA in the [0001] direction, which generates a temperature gradient in the $[01\bar{1}0]$ direction (Fig. 2(a)). To evaluate the temperature difference along this direction, we defined two regions of interest, labeled U and D (see Fig. 2(a) and the inset of Fig. 2(m)), which respectively cover the upper and lower edges of the Mn_3_Sn slab. Figure 2(m) plots the field dependence of Δ*X*, which is calculated by subtracting the average *X* values of D from those of U. The resulting Δ*X* curve exhibits a closed hysteresis loop, analogous to its AHE curve (see Fig. S2(a) within the online supplementary material). For comparison, the field dependence of the magnetization *M* is plotted in Fig. 2(m). Apart from a linear background, the Δ*X* curve is in good agreement with the *M* hysteresis loop (see Fig. S3(a) within the online supplementary material for further comparison). The Δ*X* curve obtained using this technique is similar to the temperature gradient results observed from transport measurements [38]. However, the corresponding *X* images provide visualization of the domain evolution at each point (i.e. points a–l in Fig. 2(m)) of the curve, which is beyond the capacity of transport measurements. Based on the calculated thermal gradient ∇*T* along $[01\bar{1}0]$, the AEE coefficient of Mn_3_Sn is estimated to be |ε_AEE_| ≈ 18 *μ*K m A^−1^, which is the first experimentally determined value for this material. The ANE coefficient |*S*_ANE_| of Mn_3_Sn reported by Li *et al.* [11] is about 0.5 *μ*V K^−1^ at room temperature, with a thermal conductivity κ of approximately 7.4 W K^−1^m^−1^. Using the Bridgman relation ε_AEE_ = *TS*_ANE_/κ [41], this predicts an AEE coefficient |ε_AEE_| of about 20 *μ*K m A^−1^, which is close to our measured value.

Panels (b) and (g) of Fig. 2 show the domain patterns under zero external magnetic field, which are nearly identical to those observed in panels (a) and (f) of Fig. 2 under saturation fields, confirming a spontaneous AEE at zero field whose sign can be switched by a coercive field ∼23 mT. Meanwhile, panels (d), (e), (i) and (j) of Fig. 2 reveal four intermediate states during the reversal process, showing three sizeable single-domain regions separated by two (0001) facets. These single-domain regions undergo a sharp reversal along the $[2\bar{1}\bar{1}0]$ easy axis even with field scans in 0.01 mT steps. It should be noted that, although each domain region in this sample exhibits a sharp reversal along the easy axis, the reversal behavior can vary significantly between samples (see Fig. S4 within the online supplementary material). Furthermore, the LIT imaging, as well as other imaging methods such as MOKE and ANE, can only visualize domain regions composed of a large number of magnetic octupoles and cannot resolve the spin structure of magnetic octupoles or the internal structure of domain walls.

### Imaging the in-plane magnetic reversal of Mn_3_Sn

**Figure 3.**
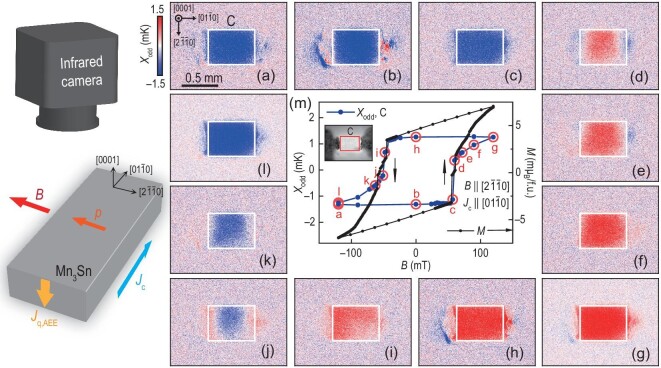
Infrared imaging of magnetic domain patterns in Mn_3_Sn during in-plane magnetic reversal. (a)–(l) The *X*_odd_ images obtained in the in-plane field-scan cycle from −120 to 120 mT along $[2\bar{1}\bar{1}0]$ at *J*_c_ = 50 mA along $[01\bar{1}0]$, where *X*_odd_ denotes the lock-in temperature modulation with the *B*-odd dependence. (m) Field dependence of *X*_odd_ on the C area of the Mn_3_Sn slab, where the plotted data were obtained by averaging the values on C, which consists of 10 800 pixels. For comparison, the field dependence of magnetization *M* is shown. Inset shows the DC infrared image of the Mn_3_Sn slab with the surface along the (0001) direction. The thickness and width of the slab are approximately 0.12 and 0.45 mm, with a charge current density of *j*_c_ = 0.9 × 10^3^ A m^−2^.

Figure 3 presents the results of the in-plane domain reversal of a Mn_3_Sn sample with the surface oriented along the (0001) direction. Panels (a)–(l) of Fig. 3 display the *X*_odd_ images obtained through an in-plane field-scan cycle ranging from −120 to 120 mT along $[2\bar{1}\bar{1}0]$ at *J*_c_ = 50 mA along $[01\bar{1}0]$. Here, *X*_odd_ = *A*_odd_ cos ϕ_odd_ refers to the lock-in temperature modulation with *B*-odd dependence, which eliminates a background primarily originating from the Peltier effect of the two contact electrodes [32], where $A_{\rm {odd}}e^{i\phi _{\rm {odd}}} = Ae^{i\phi } - (A_{\rm {max}B}e^{i\phi _{\rm {max}B}}+A_{\rm {min}B}e^{i\phi _{\rm {min}B}})/2$, and *A*_maxB_ (ϕ_maxB_) and *A*_minB_ (ϕ_minB_) represent the lock-in amplitude (phase) at the maximum positive magnetic field and minimum negative magnetic field, respectively. In this configuration, the temperature gradient of AEE is in the out-of-plane direction, resulting in the formation of corresponding heating or cold cooling regions on the surface, depending on the direction of the in-plane octupole moment. Thus, the color change from blue to red and back to blue in panels (a)–(l) of Fig. 3 corresponds to the in-plane reversal process along the $[2\bar{1}\bar{1}0]$ direction. Figure 3(m) illustrates the field dependence of the averaged *X*_odd_ on the C region (defined in Fig. 3(a); see also the inset of Fig. 3(m)), which exhibits a hysteresis loop. For comparison, the field dependence of magnetization *M* is also shown in Fig. 3(m), and we find that both dependencies are in good agreement (see Fig. S3b within the online supplementary material). By combining the *X*_odd_ images at selected points on the hysteresis (e.g. Fig. 3(d), (e), (j) and (k)), it is suggested that the in-plane reversal of this sample involves a rather gradual process of ${\boldsymbol {\mathrm{p}}}$ rotation initially in the middle and then on both sides. The results in Figs 2 and 3 demonstrate the effectiveness of AEE as a versatile tool to observe both out-of-plane and in-plane octupole domain structures.

### Imaging the in-plane and out-of-plane magnetic reversals of Mn_3_Ge

**Figure 4.**
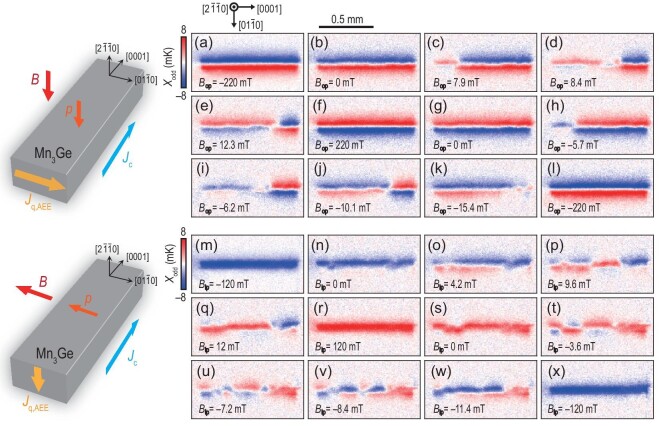
Infrared imaging of magnetic domain patterns in Mn_3_Ge during out-of-plane and in-plane magnetic reversals. (a)–(l) The *X*_odd_ images obtained in the out-of-plane field-scan cycle from −220 to 220 mT along $[2\bar{1}\bar{1}0]$ at *J*_c_ = 50 mA along [0001]. (m)–(x) The *X*_odd_ images obtained in the in-plane field-scan cycle from −120 to 120 mT along $[01\bar{1}0]$ at *J*_c_ = 50 mA along [0001]. Here *X*_odd_ denotes the lock-in temperature modulation with the *B*-odd dependence.

Figure 4 presents the results of out-of-plane and in-plane domain reversals of a Mn_3_Ge sample with the surface oriented along the $[2\bar{1}\bar{1}0]$ direction. Panels (a)–(l) of Fig. 4 display the *X*_odd_ images obtained through an out-of-plane field-scan cycle ranging from −220 to 220 mT along $[2\bar{1}\bar{1}0]$ at *J*_c_ = 50 mA along [0001]. Analysis of Fig. 4(c)–(e) and (h)–(j) indicates that the out-of-plane domain reversal process in this sample proceeds from left to right, rather than being a sharp up-to-down transition, passing through the $[01\bar{1}0]$ direction. Panels (m)–(x) of Fig. 4 display the *X*_odd_ images obtained through an in-plane field-scan cycle ranging from −120 to 120 mT along $[01\bar{1}0]$ at *J*_c_ = 50 mA along [0001]. It is evident that the in-plane reversal process also occurs from left to right and involves traversing the $[2\bar{1}\bar{1}0]$ direction. The results presented in Fig. 4 exemplify the versatility of our infrared imaging technique in studying the Mn_3_X family. The ANE coefficient |*S*_ANE_| of Mn_3_Ge reported by Tomita *et al.* [47] is about 0.35 *μ*V K^−1^ at 300 K, with a thermal conductivity κ of approximately 6.6 W K^−1^m^−1^. The AEE coefficient |ε_AEE_| of Mn_3_Ge was first measured by Xu *et al.* [38], which is about 15 *μ*K m A^−1^ at 300 K. The value we estimated by LIT is about 17 *μ*K m A^−1^, close to their value by transport measurements.

### Visualization of the magnetic memory effect

**Figure 5.**
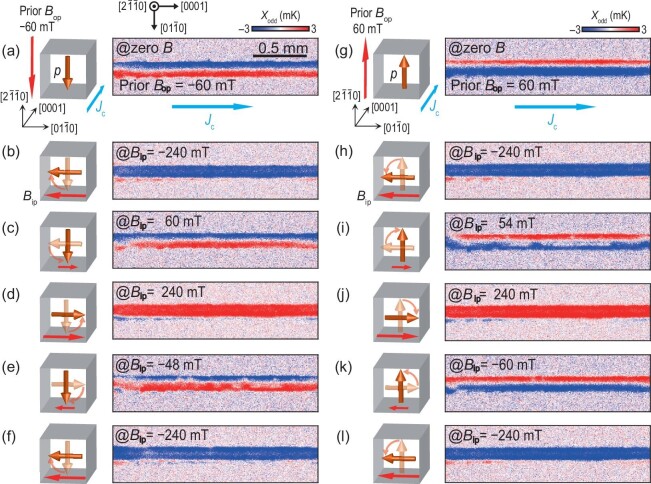
Visualization of the magnetic memory effect by the anomalous Ettingshausen effect in Mn_3_Sn. (a), (g) The initial orientation of the octupole moment and the corresponding *X*_odd_ image obtained at zero field and *J*_c_ = 50 mA along [0001], after applying a prior field *B*_op_ = −60 mT (a) and *B*_op_ = 60 mT (g) along $[2\bar{1}\bar{1}0]$. (b)–(f), (h)–(l) The out-of-plane rotation of the octupole moment during an in-plane field-scan cycle from −240 to 240 mT along $[01\bar{1}0]$, and the corresponding measured *X*_odd_ images, with the prior field *B*_op_ = −60 mT (b)–(f) and *B*_op_ = 60 mT (h)–(l). The chirality of the octupole moment rotation is found to depend on the direction of the prior field.

Figure 5 presents the visualization for the prior-field-dependent chirality of octupole rotation during an in-plane magnetic field sweep, which is due to the ‘memory effect’ in Mn_3_Sn [13]. Figure 5(a) illustrates the initial out-of-plane orientation of ${\boldsymbol {\mathrm{p}}}$ in a Mn_3_Sn slab after applying a prior field of −60 mT along $[2\bar{1}\bar{1}0]$, which is evidenced by the in-plane temperature gradient in the measured *X*_odd_ image obtained at *J*_c_ = 50 mA along [0001]. Panels (b)–(f) of Fig. 5 illustrate the out-of-plane rotations of ${\boldsymbol {\mathrm{p}}}$ during an in-plane field-scan cycle, along with the corresponding *X*_odd_ images. In (b), (d) and (f) of Fig. 5 uniform temperature distributions similar to that in Fig. 3(a) are found, indicating ${\boldsymbol {\mathrm{p}}}$ alignment parallel to the in-plane field. In (c) and (e) of Fig. 5 an in-plane temperature gradient similar to that in Fig. 5(a) is found, which means that the initial out-of-plane orientation of ${\boldsymbol {\mathrm{p}}}$ can be recovered at a low field during in-plane field sweeping. Therefore, ${\boldsymbol {\mathrm{p}}}$ shows an anti-clockwise rotation from *B*_ip_ = −240 mT to *B*_ip_ = 240 mT and a clockwise rotation from *B*_ip_ = 240 mT to *B*_ip_ = −240 mT. Panels (g)–(l) of Fig. 5 show the measured *X*_odd_ images for the in-plane field sweep after a reversed prior field, *B*_op_ = 60 mT. The thermal images show that ${\boldsymbol {\mathrm{p}}}$ is aligned parallel with the in-plane field at *B*_ip_ = ±240 mT, but has an opposite out-of-plane orientation in the low-field range compared to those in Fig. 5(c)–(e), evidencing the prior-field-dependent rotation chirality of ${\boldsymbol {\mathrm{p}}}$. In previous studies, the prior-field-dependent rotation chirality of ${\boldsymbol {\mathrm{p}}}$ in the ‘memory effect’ was proposed to understand the planar AHE and planar ANE during an in-plane magnetic field sweeping [13,48], which was not directly observed. Here the LIT measurement clarifies the microscopic origin of the ‘memory effect’ in Mn_3_Sn. Compared to MOKE that allows observation of the ${\boldsymbol {\mathrm{p}}}$ component along one direction in an optical set-up [14,16], our method offers a clear benefit for investigating 3D octupole moment dynamic processes.

## CONCLUSIONS

In summary, we have developed a new technique for visualizing the octupole domain structure in non-collinear AFMs using AEE at room temperature. AEE imaging of the domain configurations in non-collinear AFMs may well accelerate the development of future memory devices and facilitate the study of current-driven domain dynamics. Our preliminary *ab initio* calculation shows that AEE is sizable in several materials in the Mn_3_X family, indicating its wide application. Furthermore, some non-collinear antiferromagnetic materials may exhibit a small Kerr angle but a large AEE coefficient, for which this technique is particularly advantageous.

## METHODS

### Preparation of Mn_3_Sn samples

The Mn_3_Sn single crystals were grown by the flux method using Bi as the flux. Mn powder (99.95% purity), Sn granules (99.999% purity) and Bi granules (99.999% purity) were mixed in a 3:1:3 molar ratio. This mixture was loaded into an alumina crucible, sealed in a quartz ampoule under a partial argon atmosphere and heated to 1150 ^○^C in a furnace within 15 hours, maintaining this temperature for 10 hours. The assembly was then slowly cooled to 700 ^○^C at a rate of 50 ^○^C per hour, held at that temperature for 10 hours, and further gradually cooled to 300 ^○^C at a rate of 2 ^○^C per hour, maintaining it for 50 hours. Finally, the assembly was taken out of the furnace, and Mn_3_Sn crystals were separated from the flux using a centrifuge.

### Sample characterization

The LIT measurement was made by the Luxet thermo 100 system produced by Suzhou Luxet Infrared Technology Co., Ltd. The magnetization was measured using a commercial superconducting quantum interference device magnetometer (MPMS, Quantum Design). The Hall resistivity was measured using a standard four-probe geometry with a 10-mA current. Magneto-optical imaging of Mn_3_Sn crystals with $(2\bar{1}\bar{1}0)$ oriented surface plane was performed at room temperature using a Kerr microscope with a 620-nm wavelength LED (TuoTuo Technology).

### Experimental set-up

The LIT system is composed of an infrared camera, a microscope lens, a system processing unit that performs real-time Fourier analysis of detected thermal images and a source meter. Electromagnets situated underneath the infrared camera enable the measurement of thermal images under in-plane or out-of-plane magnetic fields. To prevent vibrations from interfering with the measurements, the camera and electromagnet are situated on a vibration isolation table. All Mn_3_Sn samples used in this study were unpolished and their surfaces were coated with insulating black ink for LIT measurements.

## Supplementary Material

nwad308_Supplemental_Files

## References

[1] Higo T, Kondou K, Nomoto T et al. Perpendicular full switching of chiral antiferromagnetic order by current. Nature 2022; 607: 474–9. 10.1038/s41586-022-04864-1 35859198

[2] Tsai H, Higo T, Kondou K et al. Electrical manipulation of a topological antiferromagnetic state. Nature 2020; 580: 608–13. 10.1038/s41586-020-2211-2 32350469

[3] Liu ZQ, Chen H, Wang JM et al. Electrical switching of the topological anomalous Hall effect in a non-collinear antiferromagnet above room temperature. Nat Electron 2018; 1: 172–7. 10.1038/s41928-018-0040-1

[4] Qin P, Yan H, Wang X et al. Room-temperature magnetoresistance in an all-antiferromagnetic tunnel junction. Nature 2023; 613: 485–9. 10.1038/s41586-022-05461-y 36653565

[5] Chen X, Higo T, Tanaka K et al. Octupole-driven magnetoresistance in an antiferromagnetic tunnel junction. Nature 2023; 613: 490–5. 10.1038/s41586-022-05463-w 36653566 PMC9849134

[6] Jeon KR, Hazra BK, Kim JK et al. Chiral antiferromagnetic Josephson junctions as spin-triplet supercurrent spin valves and d.c. SQUIDs. Nat Nanotechnol 2023; 18: 747–53. 10.1038/s41565-023-01336-z 36997754 PMC10359187

[7] Deng Y, Liu X, Chen Y et al. All-electrical switching of a topological non-collinear antiferromagnet at room temperature. Natl Sci Rev 2022; 10: nwac154. 10.1093/nsr/nwac154 36872930 PMC9977383

[8] Hu S, Shao DF, Yang H et al. Efficient perpendicular magnetization switching by a magnetic spin Hall effect in a noncollinear antiferromagnet. Nat Commun 2022; 13: 4447. 10.1038/s41467-022-32179-2 35915121 PMC9343665

[9] Nakatsuji S, Kiyohara N, Higo T. Large anomalous Hall effect in a non-collinear antiferromagnet at room temperature. Nature 2015; 527: 212–5. 10.1038/nature15723 26524519

[10] Ikhlas M, Tomita T, Koretsune T et al. Large anomalous Nernst effect at room temperature in a chiral antiferromagnet. Nat Phys 2017; 13: 1085–90. 10.1038/nphys4181

[11] Li X, Xu L, Ding L et al. Anomalous Nernst and Righi-Leduc effects in Mn_3_Sn: Berry curvature and entropy flow. Phys Rev Lett 2017; 119: 056601. 10.1103/PhysRevLett.119.056601 28949739

[12] Kuroda K, Tomita T, Suzuki MT et al. Evidence for magnetic Weyl fermions in a correlated metal. Nat Mater 2017; 16: 1090–5. 10.1038/nmat4987 28967918

[13] Li X, Collignon C, Xu L et al. Chiral domain walls of Mn_3_Sn and their memory. Nat Commun 2019; 10: 3021. 10.1038/s41467-019-10815-8 31289269 PMC6616569

[14] Higo T, Man H, Gopman DB et al. Large magneto-optical Kerr effect and imaging of magnetic octupole domains in an antiferromagnetic metal. Nat Photonics 2018; 12: 73–8. 10.1038/s41566-017-0086-z 29910828 PMC5997294

[15] Reichlova H, Janda T, Godinho J et al. Imaging and writing magnetic domains in the non-collinear antiferromagnet Mn_3_Sn. Nat Commun 2019; 10: 5459. 10.1038/s41467-019-13391-z 31784509 PMC6884521

[16] Uchimura T, Yoon JY, Sato Y et al. Observation of domain structure in non-collinear antiferromagnetic Mn_3_Sn thin films by magneto-optical Kerr effect. Appl Phys Lett 2022; 120: 172405. 10.1063/5.0089355

[17] Yan GQ, Li S, Lu H et al. Quantum sensing and imaging of spin–orbit-torque-driven spin dynamics in the non-collinear antiferromagnet Mn_3_Sn. Adv Mater 2022; 34: 2200327. 10.1002/adma.202200327 35322479

[18] Stöhr J, Scholl A, Regan TJ et al. Images of the antiferromagnetic structure of a NiO(100) surface by means of X-ray magnetic linear dichroism spectromicroscopy. Phys Rev Lett 1999; 83: 1862–5. 10.1103/PhysRevLett.83.1862

[19] Scholl A, Stöhr J, Lüning J et al. Observation of antiferromagnetic domains in epitaxial thin films. Science 2000; 287: 1014–6. 10.1126/science.287.5455.1014 10669407

[20] Hedrich N, Wagner K, Pylypovskyi OV et al. Nanoscale mechanics of antiferromagnetic domain walls. Nat Phys 2021; 17: 574–7. 10.1038/s41567-020-01157-0

[21] Krizek F, Reimers S, Kašpar Z et al. Atomically sharp domain walls in an antiferromagnet. Sci Adv 2022; 8: eabn3535. 10.1126/sciadv.abn3535 35353557 PMC8967221

[22] Xu J, Zhou C, Jia M et al. Imaging antiferromagnetic domains in nickel oxide thin films by optical birefringence effect. Phys Rev B 2019; 100: 134413. 10.1103/PhysRevB.100.134413

[23] Takeuchi Y, Yamane Y, Yoon JY et al. Chiral-spin rotation of non-collinear antiferromagnet by spin-orbit torque. Nat Mater 2021; 20: 1364–70. 10.1038/s41563-021-01005-3 33986515

[24] Safeer CK, Jué E, Lopez A et al. Spin-orbit torque magnetization switching controlled by geometry. Nat Nanotechnol 2015; 11: 143–6. 10.1038/nnano.2015.252 26551017

[25] Tang M, Shen K, Xu S et al. Bulk spin torque-driven perpendicular magnetization switching in *L*1_0_ FePt single layer. Adv Mater 2020; 32: 2002607. 10.1002/adma.202002607 32596934

[26] Liu L, Zhou C, Shu X et al. Symmetry-dependent field-free switching of perpendicular magnetization. Nat Nanotechnol 2021; 16: 277–82. 10.1038/s41565-020-00826-8 33462431

[27] Wang Y, Taniguchi T, Lin PH et al. Time-resolved detection of spin-orbit torque switching of magnetization and exchange bias. Nat Electron 2022; 5: 840–8. 10.1038/s41928-022-00870-3

[28] Xu J, Xia J, Zhang X et al. Exchange-torque-triggered fast switching of antiferromagnetic domains. Phys Rev Lett 2022; 128: 137201. 10.1103/PhysRevLett.128.137201 35426702

[29] Wu M, Isshiki H, Chen T et al. Magneto-optical Kerr effect in a non-collinear antiferromagnet Mn_3_Ge. Appl Phys Lett 2020; 116: 132408. 10.1063/1.5143959

[30] Uchida Ki, Daimon S, Iguchi R et al. Observation of anisotropic magneto-Peltier effect in nickel. Nature 2018; 558: 95–9. 10.1038/s41586-018-0143-x 29785052

[31] Wang J, Takahashi YK, Uchida Ki. Magneto-optical painting of heat current. Nat Commun 2020; 11: 2. 10.1038/s41467-019-13799-7 31911599 PMC6946696

[32] Breitenstein O, Warta W, Langenkamp M. Lock-In Thermography: Basics and Use for Evaluating Electronic Devices and Materials, 2nd edn. Berlin: Springer, 2010. 10.1007/978-3-642-02417-7

[33] Seki T, Iguchi R, Takanashi K et al. Visualization of anomalous Ettingshausen effect in a ferromagnetic film: direct evidence of different symmetry from spin Peltier effect. Appl Phys Lett 2018; 112: 152403. 10.1063/1.5022759

[34] Daimon S, Iguchi R, Hioki T et al. Thermal imaging of spin Peltier effect. Nat Commun 2016; 7: 13754. 10.1038/ncomms13754 27941953 PMC5159862

[35] Suzuki MT, Koretsune T, Ochi M et al. Cluster multipole theory for anomalous Hall effect in antiferromagnets. Phys Rev B 2017; 95: 094406. 10.1103/PhysRevB.95.094406

[36] Chen H, Niu Q, MacDonald A. Anomalous hall effect arising from noncollinear antiferromagnetism. Phys Rev Lett 2014; 112: 017205. 10.1103/PhysRevLett.112.017205 24483927

[37] Yang H, Sun Y, Zhang Y et al. Topological Weyl semimetals in the chiral antiferromagnetic materials Mn_3_Ge and Mn_3_Sn. New J Phys 2017; 19: 015008. 10.1088/1367-2630/aa5487

[38] Xu L, Li X, Lu X et al. Finite-temperature violation of the anomalous transverse Wiedemann-Franz law. Sci Adv 2020; 6: eaaz3522. 10.1126/sciadv.aaz3522 32494640 PMC7182422

[39] Chen T, Tomita T, Minami S et al. Anomalous transport due to Weyl fermions in the chiral antiferromagnets Mn_3_X, X=Sn, Ge. Nat Commun 2021; 12: 572. 10.1038/s41467-020-20838-1 33495448 PMC7835387

[40] Nagamiya T, Tomiyoshi S, Yamaguchi Y. Triangular spin configuration and weak ferromagnetism of Mn_3_Sn and Mn_3_Ge. Solid State Commun 1982; 42: 385–8. 10.1016/0038-1098(82)90159-4

[41] Bridgman PW . The connections between the four transverse galvanomagnetic and thermomagnetic phenomena. Phys Rev 1924; 24: 644–51. 10.1103/PhysRev.24.644

[42] Miura A, Sepehri-Amin H, Masuda K et al. Observation of anomalous Ettingshausen effect and large transverse thermoelectric conductivity in permanent magnets. Appl Phys Lett 2019; 115: 222403. 10.1063/1.5131001

[43] Seki T, Miura A, Uchida Ki et al. Anomalous Ettingshausen effect in ferrimagnetic Co-Gd. Appl Phys Express 2019; 12: 023006. 10.7567/1882-0786/aafb5a

[44] Duan TF, Ren WJ, Liu WL et al. Magnetic anisotropy of single-crystalline Mn_3_Sn in triangular and helix-phase states. Appl Phys Lett 2015; 107: 082403. 10.1063/1.4929447

[45] Li X, Zhu Z, Behnia K. A monomaterial Nernst thermopile with hermaphroditic legs. Adv Mater 2021; 33: 2100751. 10.1002/adma.202100751 33844874

[46] Li X, Xu L, Zuo H et al. Momentum-space and real-space Berry curvatures in Mn_3_Sn. SciPost Phys 2018; 5: 063. 10.21468/SciPostPhys.5.6.063

[47] Tomita T, Minami S, Ikhlas M et al. Anomalous transport properties of the antiferromagnetic Weyl semimetals Mn_3_X (X = Sn, Ge). J Phys Conf Ser 2022; 2164: 012065. 10.1088/1742-6596/2164/1/012065

[48] Xu L, Li X, Ding L et al. Planar Hall effect caused by the memory of antiferromagnetic domain walls in Mn_3_Ge. Appl Phys Lett 2020; 117: 222403. 10.1063/5.0030546

