# Supplementary material for: Infrared imaging of magnetic octupole domains in non-collinear antiferromagnets
Source: Natl Sci Rev. 2023 Dec 4;11(6):nwad308. doi: 10.1093/nsr/nwad308 (PMC11650872; doi:10.1093/nsr/nwad308)
Supplement: nwad308_Supplemental_Files [file nwad308_supplemental_files.zip › NSR_supplemental_file.docx]

Supplementary Information for

**Infrared Imaging of Magnetic Octupole Domains in**

**Non-collinear Antiferromagnets**

Peng Wang,^1,2,†^ Wei Xia,^3,4,†^ Jinhui Shen,^1,5^ Yulong Chen,^1,5^ Wenzhi Peng,^1,5^ Jiachen Zhang,^1,5^ Haolin Pan,^1,5^ Xuhao Yu,^1,5^ Zheng Liu,^5,6^ Yang Gao,^5,6^ Qian Niu,^5,6^ Zhian Xu,^3^ Hongtao Yang,^7^ Yanfeng Guo,^3,4,*^ and Dazhi Hou^1,5,*^

*^1^ ICQD, School of Emerging Technology, University of Science and Technology of China, Hefei 230026, China*

*^2^ College of Mathematics and Physics, Qingdao University of Science and Technology, Qingdao 266061, China*

^3^ *School of Physical Science and Technology, ShanghaiTech University, Shanghai 201210, China*

^4^ *ShanghaiTech Laboratory for Topological Physics, Shanghai 201210, China*

*^5^ Department of Physics, University of Science and Technology of China, Hefei, Anhui 230026, China*

^6^ *CAS Key Laboratory of Strongly-Coupled Quantum Matter Physics, University of Science and Technology of China, Hefei, Anhui 230026, China*

*^7^ Xi'an Institute of Optics and Precision Mechanics of Chinese Academy of Sciences, Xi'an, Shanxi 710119, P.R.China*

^†^ These authors contributed equally to this work.

^*^ Correspondence and requests for materials should be addressed to Y.F.G., or D.H.

^*^ E-mail: guoyf@shanghaitech.edu.cn, dazhi@ustc.edu.cn

**Table of Contents**

**Section S1.** MOKE images of Mn_3_Sn

**Section S2.** The AHE and the optical image of the Mn_3_Sn sample in Fig. 2

**Section S3.** Compare the *X* curves to magnetization *M*

**Section S4.** The out-of-plane AEE results of the same Mn_3_Sn sample in Fig. 4

**Section S5.** Movie of the out-of-plane magnetic reversal process in Mn_3_Sn

**Section S6.** The definition of the anomalous Ettingshausen coefficient

**Section S1. MOKE images of Mn_3_Sn**

Figure S1 shows the MOKE images of Mn_3_Sn. Kerr signal can not be detected probably due to surface oxidization and roughness. MOKE images of a 0.7-nm-thick Co film with perpendicular magnetic anisotropy (PMA) under the same set-up is also shown for comparison.


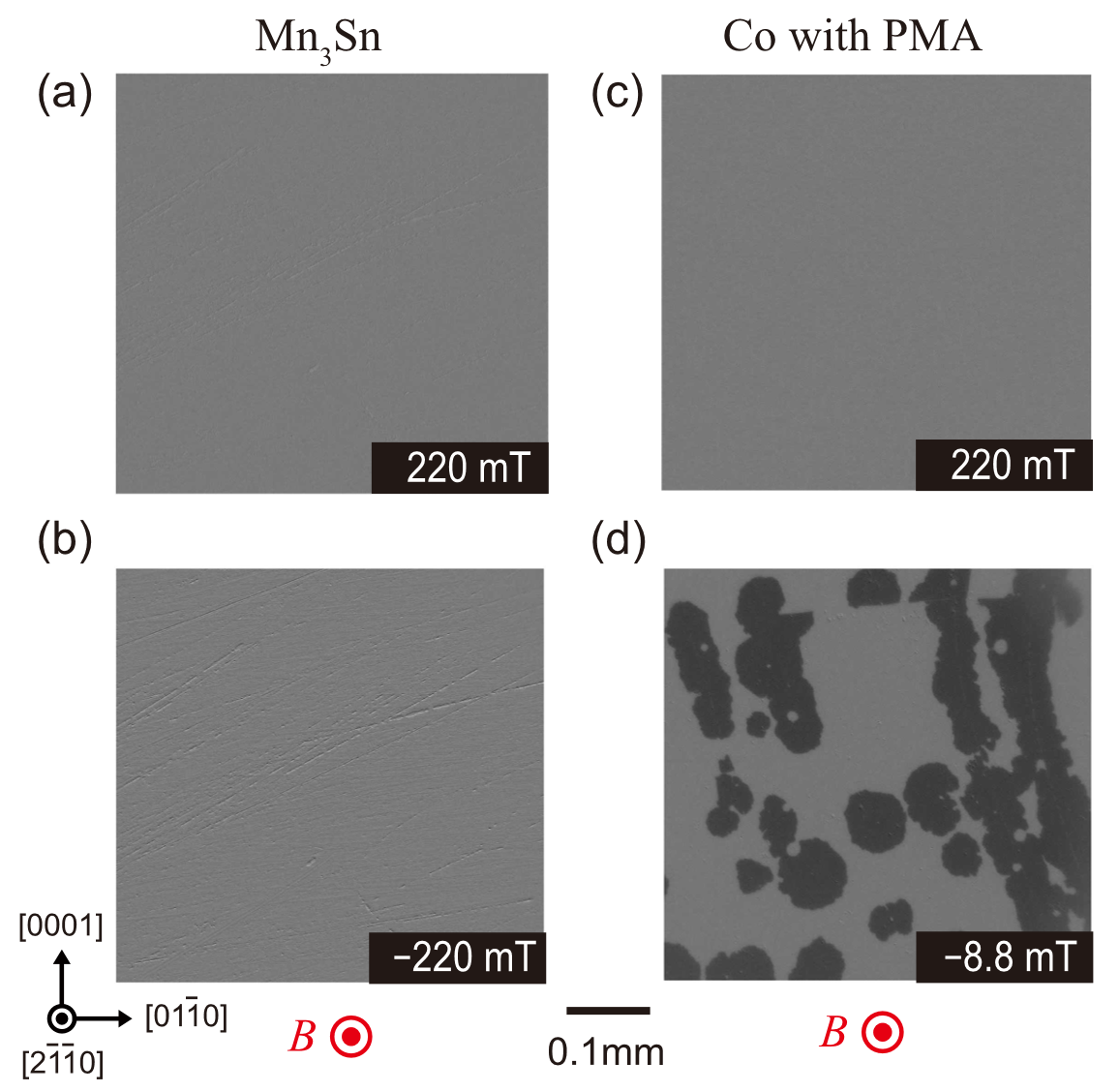


**Supplementary Fig. S1 | MOKE images of Mn_3_Sn. a**,**b**, MOKE images of Mn_3_Sn obtained at an out-of-plane field *B* = 220 mT (**a**) and *B* = −220 mT (**b**). **c**,**d**, MOKE images of a 0.7-nm-thick Co film with perpendicular magnetic anisotropy (PMA) for comparison obtained at *B* = 220 mT (**c**) and *B* = −8.8 mT (**d**). Grey and black regions correspond to positive and negative values of the MOKE signal.

**Section S2. The AHE and the optical image of the Mn_3_Sn sample in Fig. 2**

Figure S2(a) shows the measured anomalous Hall effect (AHE) signal of the same Mn_3_Sn sample in Fig. 1 and Fig. 2 in the main text. Figure S2(b) shows the optical image of this sample, which is covered with insulating black ink for lock-in thermography measurements.


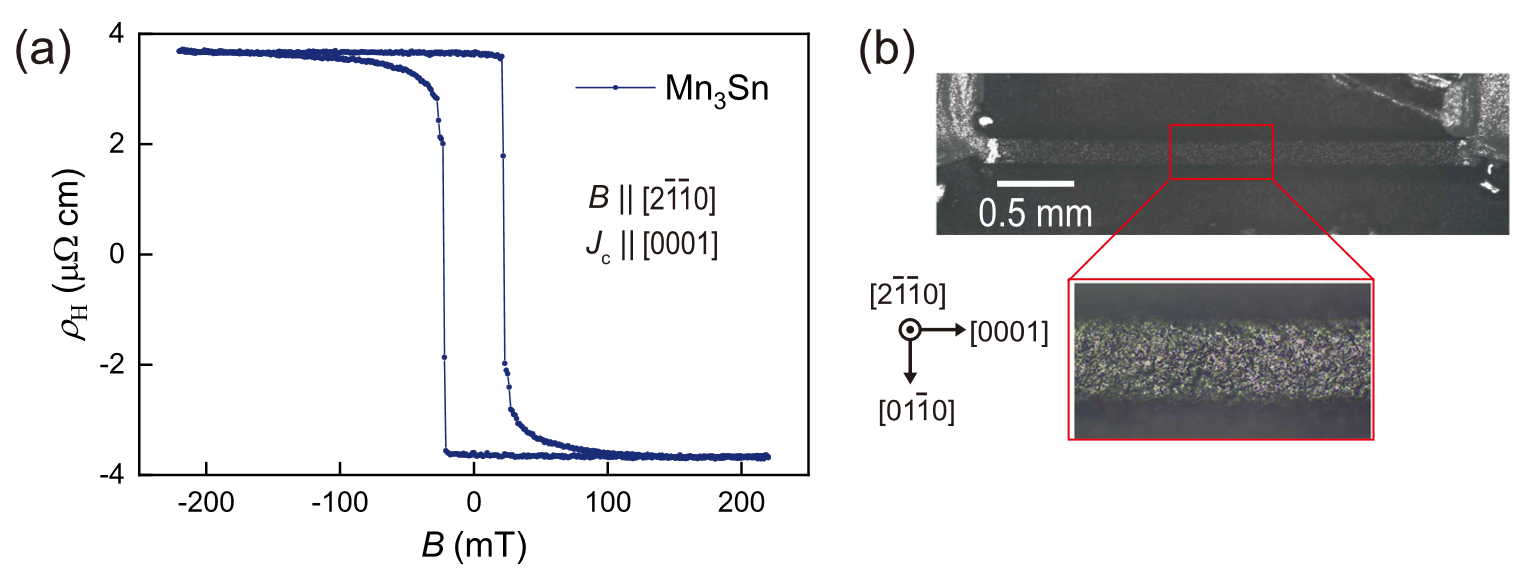


**Supplementary Fig. S2 | The AHE (a) and the** **optical image (b) of the Mn_3_Sn sample in Fig. 2.**

**Section S3. Compare the *X* curves to magnetization *M***


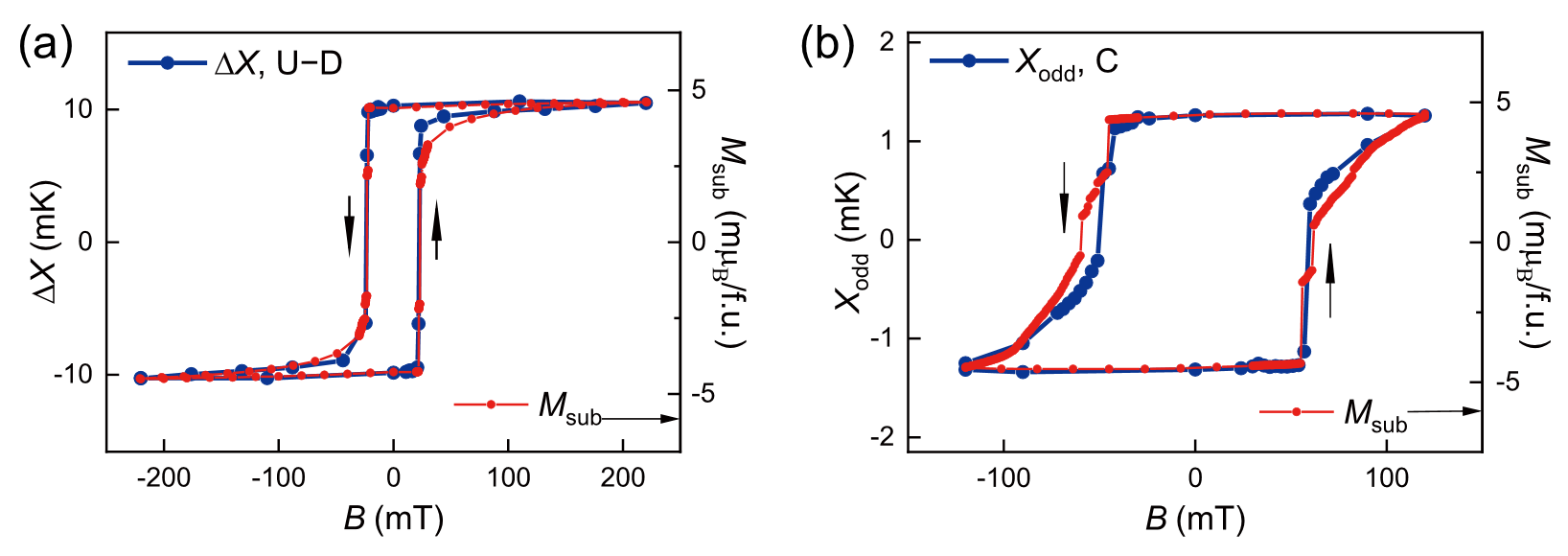


**Supplementary Fig. S3 | Compare the ∆*X* (or *X*_odd_) curves in Fig. 2 and Fig. 3 to magnetization *M* subtracted by a linear function.**

**Section S4. The out-of-plane AEE results of the same Mn_3_Sn sample in Fig. 4**

Figure S4 shows the *X*_odd_ images and the corresponding ∆*X*_odd_ curve obtained in the out-of-plane field-scan cycle of the same Mn_3_Sn sample in Fig. 4 in the main text.


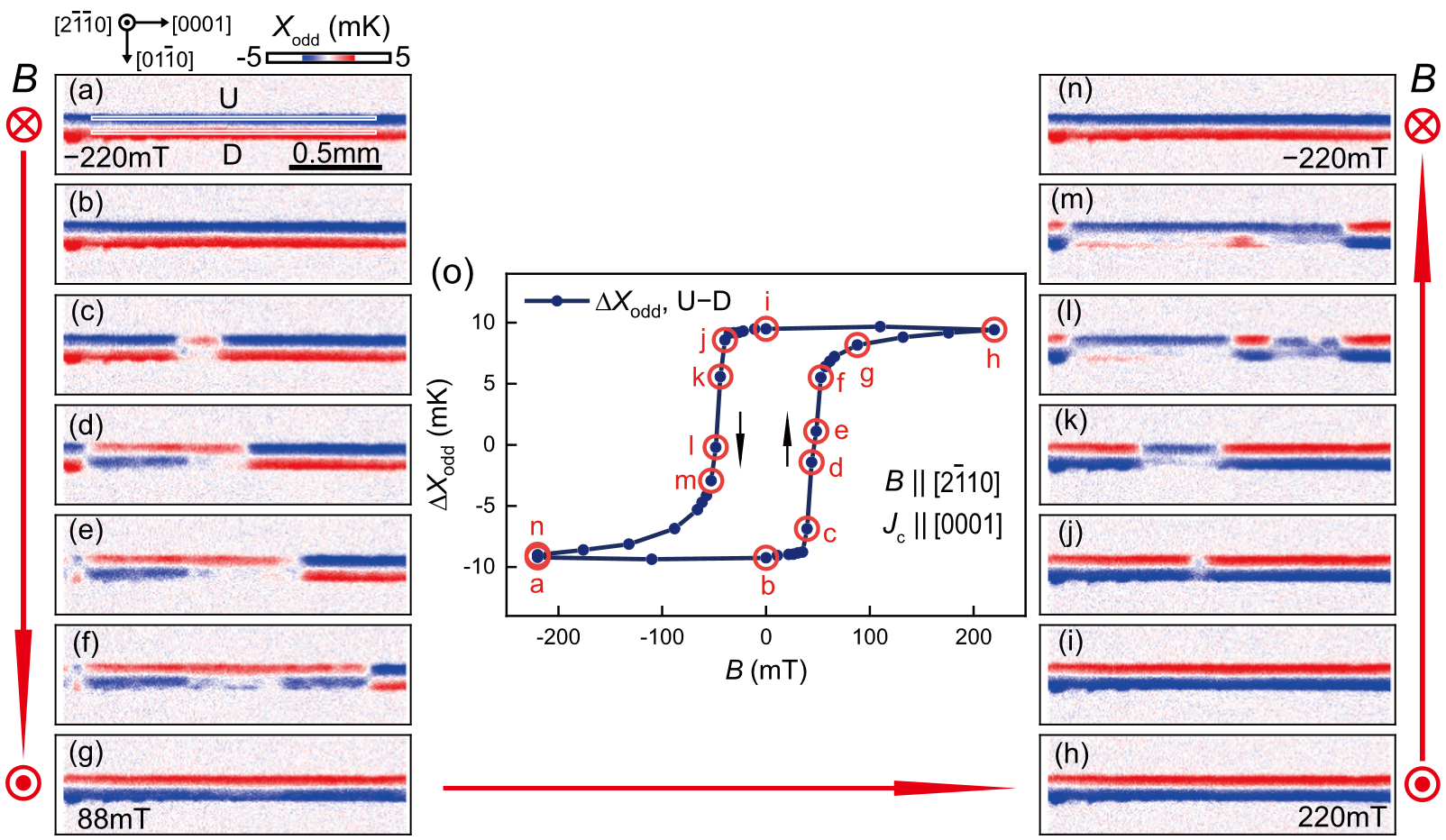


**Supplementary Fig. S4 | The out-of-plane AEE results of the same Mn_3_Sn sample in Fig. 4.** **a**-**n**, *X*_odd_ images obtained in the out-of-plane field-scan cycle from −220 mT to 220 mT along [20] at *J*_c_ = 50 mA along [0001], where *X*_odd_ denotes the lock-in temperature modulation with the *B*-odd dependence. **o**, Field dependence of ∆*X*_odd_ calculated from the U and D areas of the Mn_3_Sn slab, where the plotted data were obtained by subtracting the average values on D from U.

**Section S5. Movie of the out-of-plane magnetic reversal process in Mn_3_Sn**

**Supplementary Movie S1.** The movie of the out-of-plane magnetic reversal process under a more precise magnetic field scanning with a step of 0.01 mT for the same Mn_3_Sn sample in Fig. 2 in the main text.

**Section S6.** **The definition of the anomalous Ettingshausen coefficient**

There are two prevalent definitions for the anomalous Ettingshausen effect (AEE) coefficient in published literatures. The first defines the AEE coefficient as $\varepsilon_{\mathrm{AEE}}=\nabla_{y}T/j_{x}$ [1,2], where $\nabla_{y}T$ is the transverse thermal gradient generated by the AEE, and $j_{x}$ is the longitudinal current density. This definition is equivalent to the one used in our study, ${\nabla T}_{\mathrm{AEE}}=\varepsilon_{\mathrm{AEE}}(\mathbf{j}_{c}\times\mathbf{p})$. Under this definition, the Bridgman relation, linking the AEE coefficient $\varepsilon_{\mathrm{AEE}}$ and the anomalous Nernst effect (ANE) coefficient $S_{\mathrm{ANE}}$, is expressed as $\varepsilon_{\mathrm{AEE}}=S_{\mathrm{ANE}}T/\kappa$ [3,4], with $T$ being the absolute temperature and $\kappa$ being the thermal conductivity.

The second definition characterizes the AEE coefficient as$\mathbf{j}_{q,AEE}=\Pi_{\mathrm{AEE}}(\mathbf{j}_{c}\times\mathbf{m})$ [5,6], where $\mathbf{j}_{q,AEE}$, $\Pi_{\mathrm{AEE}}$, $\mathbf{j}_{c}$, and $\mathbf{m}$ denote the heat current density generated by the AEE, the AEE coefficient, the charge current density, and the unit vector of magnetization $\mathbf{M}$, respectively. Under this definition, the Brigman relation is $\Pi_{\mathrm{AEE}}=S_{\mathrm{ANE}}T$, which exhibits a formal similarity to the Kelvin relation $\Pi=ST$, with $\Pi$ being the Peltier coefficient and *S* being the Seebeck coefficient.

Both definitions of the AEE coefficient are commonly employed, differing only in terms of the thermal conductivity $\kappa$. Since we did not directly measure thermal conductivity, we have adopted the first definition in our work.

**References:**

1. Behnia K. *Fundamentals of Thermoelectricity*. Oxford University Press, 2015.
2. Xu L, Li X, Lu X *et al.* Finite-temperature violation of the anomalous transverse Wiedemann-Franz law. *Sci Adv* 2020; **6**: eaaz3522.
3. Bridgman PW. The Connections between the Four Transverse Galvanomagnetic and Thermomagnetic Phenomena. *Phys Rev* 1924;**24**:644–51.
4. Callen HB. The Application of Onsager’s Reciprocal Relations to Thermoelectric, Thermomagnetic, and Galvanomagnetic Effects. *Phys Rev* 1948;**73**:1349–58.
5. Seki T, Iguchi R, Takanashi K *et al.* Relationship between anomalous Ettingshausen effect and anomalous Nernst effect in an FePt thin film. *J Phys D Appl Phys* 2018;**51**:254001.
6. Miura A, Sepehri-Amin H, Masuda K *et al.* Observation of anomalous Ettingshausen effect and large transverse thermoelectric conductivity in permanent magnets. *Appl Phys Lett* 2019;**115**:222403.
